# Are we prepared to monitor and prevent patient self-inflicted lung injury (P-SILI) during mechanical ventilation in pediatric patients?

**DOI:** 10.31744/einstein_journal/2025CE1522

**Published:** 2025-02-07

**Authors:** João Marcos Feliciano de Souza, Marcelo Britto Passos Amato, Eduardo Leite Vieira Costa, Eduardo Juan Troster

**Affiliations:** 1 Faculdade Israelita de Ciências da Saúde Albert Einstein Hospital Israelita Albert Einstein São Paulo SP Brazil Faculdade Israelita de Ciências da Saúde Albert Einstein, Hospital Israelita Albert Einstein, São Paulo, SP, Brazil.; 2 Universidade de São Paulo Faculdade de Medicina Hospital das Clínicas São Paulo SP Brazil Hospital das Clínicas, Faculdade de Medicina, Universidade de São Paulo, São Paulo, SP, Brazil.

Dear Editor,

Acute respiratory failure (ARF) is a leading cause of pediatric hospitalizations, and invasive mechanical ventilation (IMV) is an essential support in severe cases.^(1)^ Data obtained through expiratory and inspiratory pause maneuvers on mechanical ventilators during spontaneous breathing in adult IMV patients have been shown to aid prevention of patient self-inflicted lung injury (P-SILI).^(2,3)^

**Figure 1:**
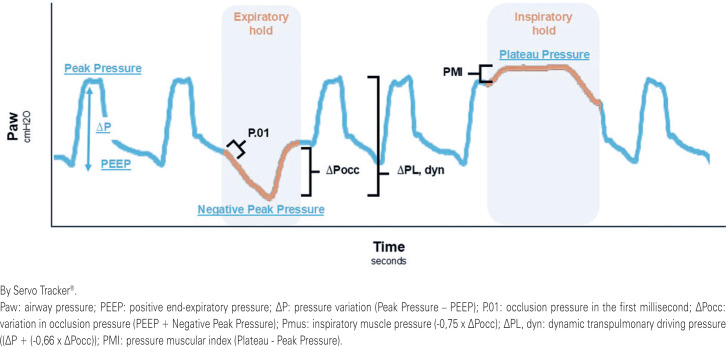
Mechanical ventilation chart taken during pressure support ventilation

The scarcity of pediatric-specific data complicates the monitoring and prevention of comorbidities associated with P-SILI. Understanding and incorporating measures (Figure 1) such as occlusion pressure in the first millisecond (P.01), variation in occlusion pressure (ΔPocc), inspiratory muscle pressure (Pmus), Pressure Muscular Index (PMI), and dynamic transpulmonary driving pressure (ΔPL, dyn) into routine ventilatory care may represent a significant step toward reducing morbidity and mortality in pediatric patients.^(4,5)^

Therefore, there is an urgent need to assess whether we are prepared to monitor and prevent P-SILI by adopting protective strategies based on pulmonary mechanics.
